# PHPB Attenuated Cognitive Impairment in Type 2 Diabetic KK-Ay Mice by Modulating SIRT1/Insulin Signaling Pathway and Inhibiting Generation of AGEs

**DOI:** 10.3390/ph16020305

**Published:** 2023-02-15

**Authors:** Jiang Li, Shaofeng Xu, Ling Wang, Xiaoliang Wang

**Affiliations:** State Key Laboratory of Bioactive Substances and Functions of Natural Medicines, Institute of Materia Medica, Chinese Academy of Medical Sciences and Peking Union Medical College, Beijing 100050, China

**Keywords:** potassium 2-(1-hydroxypentyl)-benzoate (PHPB), diabetic encephalopathy (DE), advanced glycation end products (AGEs), insulin signaling pathway, oxidative stress

## Abstract

Diabetes mellitus (DM) has been recognized as an increased risk factor for cognitive impairment, known as diabetic encephalopathy (DE). Hyperglycemia and insulin resistance are the main initiators of DE, which is related to the accumulation of advanced glycation end products (AGEs). Potassium 2-(1-hydroxypentyl)-benzoate (PHPB), a derivative of 3-n-butylphthalide (dl-NBP), has emerged various properties including improved mitochondrial function, antioxidant, anti-neuroinflammation, and neuroprotective effects. The present study aimed to investigate the neuroprotective effect of PHPB against AGEs accumulation in type 2 diabetic KK-Ay mice model with DE and further explore the underlying mechanisms. The results showed that PHPB markedly ameliorated the spatial learning ability of KK-Ay mice in the Morris water maze and decreased AD-like pathologic changes (Tau hyperphosphorylation) in the cortex. Furthermore, we found that PHPB treatment significantly reduced AGEs generation via up-regulation of glyoxalase-1 (GLO1) protein and enhancement of methylglyoxal (MG) trapping, while there was no obvious difference in levels of glucose in plasma or brain, contents of total cholesterol (TC), triglycerides (TG), and plasma insulin. Also, PHPB treatment improved the insulin signaling pathway by increasing sirtuin1 (SIRT1) deacetylase activity and attenuated oxidative stress evidenced by elevating glucose-6-phosphate dehydrogenase (G-6-PD) protein expression, promoting the production of reduced glutathione (GSH) and reduced nicotinamide adenine dinucleotide phosphate (NADPH), restoring mitochondrial membrane potential, increasing adenosine triphosphate (ATP) generation, and reducing malondialdehyde (MDA) levels in the brain. Taken together, PHPB exhibited a beneficial effect on DE, which involved modulating the SIRT1/insulin signaling pathway and reducing oxidative stress by inhibiting the generation of AGEs.

## 1. Introduction

Diabetes mellitus (DM) is one of the most common metabolic diseases characterized by hyperglycemia. According to the International Diabetes Federation, the global diabetes prevalence in 2021 is estimated to be 10.5% in 20–79 years old (536.6 million people), and type 2 diabetes mellitus (T2DM) accounts for almost 90% of all diabetes worldwide. It is predicted that 12.2% (783.2 million) of the population will have diabetes by 2045. People living with diabetes are at risk of developing a number of serious and life-threatening complications, leading to a serious threat to global health and a serious burden to families and society [1]. In particular, there is an accumulating body of evidence suggesting that DM has an increased risk of cognitive dysfunction and/or neurodegeneration leading to dementia, i.e., diabetic encephalopathy (DE); 29% of people with type 2 diabetes will eventually suffer from severe cognitive decline and neurodegeneration [2,3,4]. There is a growing body of evidence implicating that advanced glycation end products (AGEs) play a key role in the induction and aggravation of brain dysfunction in DE [5,6,7]. AGEs are a heterogeneous group of molecules and are formed by nonenzymatic glycation that occurs between lipids, proteins, reduced sugars, or nucleic acids through the Maillard reaction [7]. Methylglyoxal (MG) is a major precursor of AGEs, and the accumulation of MG in the body accelerates the generation of AGEs [8]. The accumulation of AGEs can lead to an increase of amyloid plaques and neurofibrillary tangles, hyperphosphorylation of tau, autoimmunity/inflammation, mitochondrial dysfunction/reactive oxygen species (ROS), and disrupt normal cell signaling to cause insulin resistance via down-regulation of the sirtuin1 (SIRT1) protein, which may contribute to the development of neurodegenerative diseases [8,9,10]. In addition, glyoxalase-1 (GLO-1), a main enzyme involved in MG detoxification, can promote the clearance of α-carbonyl aldehydes, such as MG, and inhibit the generation of AGEs [10]. Therefore, enhancement of MG clearance and, subsequently, inhibition of AGEs generation may be important therapeutic targets for the treatment of DE.

Potassium 2-(1-hydroxypentyl)-benzoate (PHPB), a derivative of 3-n-butylphthalide (dl-NBP) isolated from the seeds of *Apium graveolens* Linn or Chinese celery, is in phase II–III clinical trials for the treatment of cerebral ischemic stroke in China [11]. Moreover, PHPB has been verified effective in the treatment of Alzheimer’s disease (AD) by attenuating amyloid and tau protein pathologies in APP/PS1 transgenic mice, promoting mitochondrial function, antioxidant, anti-neuroinflammation in chronic cerebral hypoperfused rats, β-amyloid protein 1–40 (Aβ_1-40_)-intracerebroventricular infused rats, APP/PS1-transgenic mice, and lipopolysaccharide-induced inflammatory mice [12]. Recently, our research also proved that PHPB could significantly improve cognitive function in type 2 diabetic KK-Ay mice via elevation of neurotrophic factor and vesicular glutamate transporter 1 (vGLUT1), inhibition of neuroinflammatory, and modulation of PI3K/Akt/GSK-3β signaling pathway [13,14]. However, the precise mechanism of PHPB in the treatment of DE is not fully understood. In the present study, we investigate the beneficial effects of PHPB on DE and further characterize the potential molecular mechanisms focused on modulation of the SIRT1/insulin signaling pathway and oxidative stress via inhibiting the generation of AGEs in type 2 diabetic KK-Ay mice. In addition, a previous study has shown that rosiglitazone could significantly inhibit AGEs production in the hippocampus and cortex of C57BL/KsJ-db/db (db/db) mice [15]. Therefore, rosiglitazone served as a positive control in our study.

## 2. Results

### 2.1. The Effects of PHPB on the Metabolic Features and Cognitive Function in KK-Ay Mice

**Figure 1:**
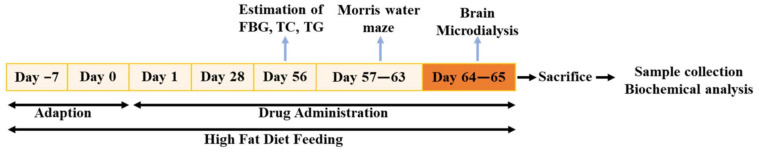
Schematic diagram of the experimental design. (Note: FBG: Fasting Blood Glucose, TC: Total Cholesterol; TG: Triglycerides).

**Figure 2:**
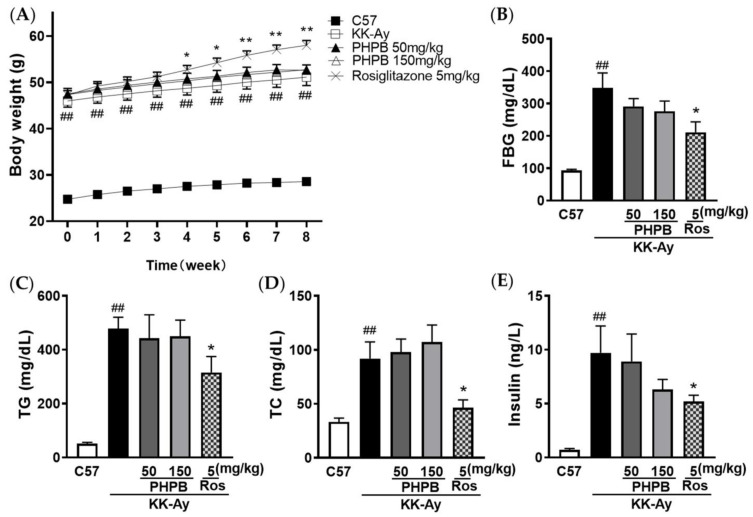
Effects of PHPB on body weight and metabolic features in mice. KK-Ay mice were treated with PHPB (50 or 150 mg/kg) or rosiglitazone (5 mg/kg) for two months by oral gavage. (**A**) Body weight, (**B**) Fasting blood glucose (FBG), (**C**) Plasma triglycerides (TG), (**D**) Plasma total cholesterol (TC), and (**E**) Plasma insulin. Values are represented as mean ± SEM. *n* = 10 for C57 group, *n* = 19 for KK-Ay group, *n* = 17 for PHPB 50 mg/kg group, *n* = 17 for PHPB 100 mg/kg group, *n* = 16 for rosiglitazone group. ## *p* < 0.01 versus C57 group; * *p* < 0.05, ** *p* < 0.01 versus KK-Ay group.

**Figure 3:**
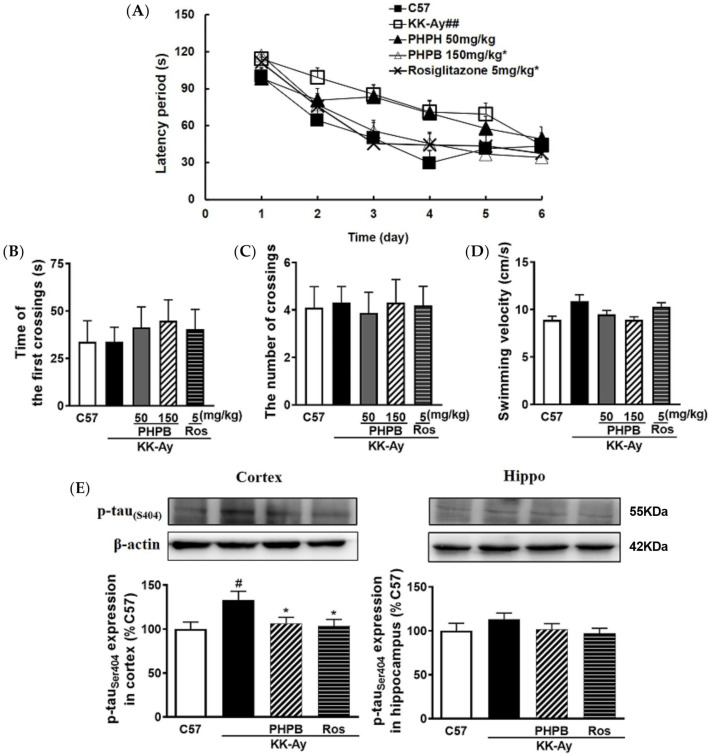
PHPB attenuated cognitive impairment in type 2 diabetic KK-Ay mice. KK-Ay mice were treated with PHPB (50 mg/kg or 150 mg/kg or rosiglitazone (Ros, 5 mg/kg) for two months by oral gavage. The performance of the Morris water maze was tested among all groups. (**A**) Spatial learning performance was analyzed by escape latency for mice to locate the hidden platform during the 6-days acquisition training. In the probe trial test on the seventh day, (**B**) the first latency time and (**C**) the times of crossings over the original platform location, and (**D**) the swimming velocity were recorded to evaluate the spatial memory maintenance/movement ability, *n* = 10–19 for each group. Furthermore, (**E**) the expression of classical hallmarks of AD, p-Tau _(S404)_ protein, was detected in the cortex and hippocampus by western blotting, *n* = 8 for each group. And then, quantitative analysis of the expression of the p-Tau protein is presented as fold change relative to C57 mice. The data are presented as means ± SEM. # *p* < 0.05, ## *p* < 0.01 versus C57 group; * *p* < 0.05 versus KK-Ay group.

A schematic diagram of the experimental design was shown in Figure 1. At the end of drug administration, 5-month-old KK-Ay mice displayed severe obesity along with distinct characteristics of T2DM (Figure 2) and cognitive deficits (Figure 3), compared with age-matched C57BL/6J mice. During PHPB (50 or 150 mg/kg) treatment for two months, there were no significant changes in the body weight and metabolic index relative to those of vehicle-treated KK-Ay mice (Figure 2), with a downtrend of the FBG level by 21% (*p* > 0.05) and insulin level by 35% (*p* > 0.05), respectively (Figure 2B,E). Rosiglitazone (5 mg/kg) intervention could significantly increase body weight (10%, *p* < 0.01) and decrease the levels of FBG (39%, *p* < 0.05), TC (50%, *p* < 0.05), TG (34%, *p* < 0.05), and insulin (47%, *p* < 0.05), respectively, in KK-Ay mice (Figure 2).

In the Morris water maze, the escape latency to find the hidden platform was used to evaluate the spatial learning and memory ability of the mice. As shown in Figure 3A, the shortened escape latency during the 6-days acquisition training was observed in PHPB treated group (*p* < 0.05), indicating that PHPB (150 mg/kg) was effective in rescuing spatial learning deficit in KK-Ay mice. Rosiglitazone (5 mg/kg) treatment also significantly reduced the time to find the platform (*p* < 0.05). Moreover, the effects of PHPB and rosiglitazone on spatial memory deficit in KK-Ay mice were also investigated in the probe trial test. There was no significant difference among all groups in first crossing time and passing times of the original platform location, and swimming velocity (Figure 3B–D).

Furthermore, accumulated evidence suggests hyperphosphorylated Tau is acted as a histopathological marker of AD [16]. In the present study, the expression of p-Tau in KK-Ay mice was markedly increased by 33% (*p* < 0.05) in the cortex relative to that of C57BL/6J mice, whereas treatments with PHPB (150 mg/kg) or rosiglitazone could obviously reduce its expression by 20% (*p* < 0.05) or 22% (*p* < 0.05), respectively (Figure 3E).

Above data indicated that PHPB has the potential for neuroprotection in DE.

### 2.2. Effects of PHPB on Glucose Levels and ATP Contents in Brains of KK-Ay Mice

**Figure 4:**
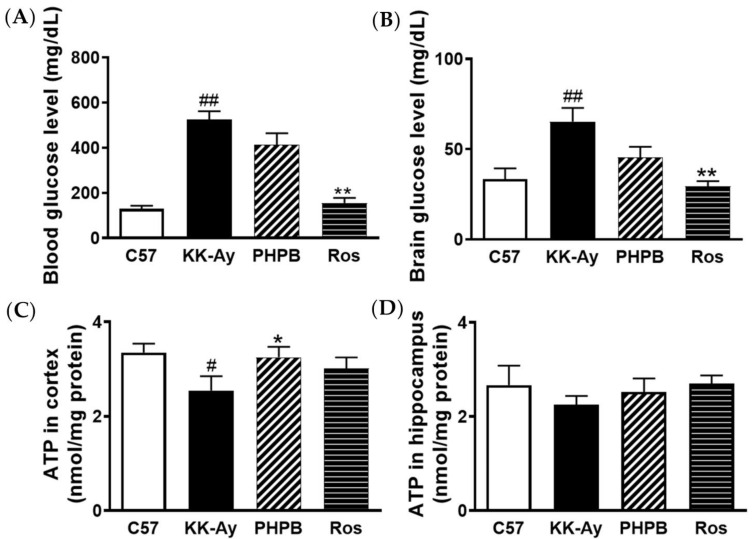
PHPB increased ATP production in the cortex of KK-Ay mice without any obvious differences in glucose levels. (**A,B**) Quantification of blood-glucose and cerebral-glucose using a micro-dialysis method in mice treated with PHPB (150 mg/kg) or rosiglitazone (Ros, 5 mg/kg) for two months, N = 7–10 for each group. (**C,D**) Detection of the ATP contents in the cortex and hippocampus by firefly luciferase method, *n* = 8 for each group. The data are presented as means ± SEM. # *p* < 0.05 versus C57 group. # *p* < 0.05, ## *p* < 0.01 versus C57 group; * *p* < 0.05, ** *p* < 0.01 versus KK-Ay group.

Dysfunction of brain glucoregulation might lead to cognitive impairment [17]. We checked the effects of PHPB on glucose levels and ATP production in KK-Ay mice. The results showed that the glucose levels in the blood and brain of KK-Ay mice were distinctly increased by 313% (*p* < 0.01) and 106% (*p* < 0.01), respectively, compared with those of C57BL/6J mice, while ATP production in the cortex was dramatically decreased by 24% (*p* < 0.05). After administration of PHPB (150 mg/kg), there were no significant differences in blood glucose level and cerebral-glucose content of the KK-Ay mice, but the production of ATP in the cortex obviously increased by 28% (*p* < 0.05). Moreover, rosiglitazone could suppress blood glucose level by 70% (*p* < 0.01) and cerebral-glucose content by 55% (*p* < 0.01), respectively, and increase cortical ATP production by 18% (*p* > 0.05) in KK-Ay mice (Figure 4).

### 2.3. Effects of PHPB on Accumulation of AGEs and MG and Expression of GLO1 in KK-Ay Mice

**Figure 5:**
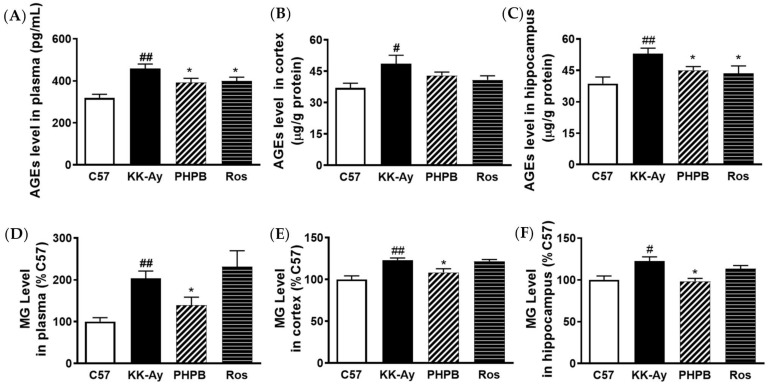
PHPB decreased the generation of AGEs or MG in the plasma and brain of KK-Ay mice. (**A**–**C**) Quantification of AGEs levels in plasma, cortex, and hippocampus by ELISA kits, and its levels were normalized to total protein concentration in the tissue samples of mice treated by PHPB (150 mg/kg) or rosiglitazone (Ros, 5 mg/kg) for two months, *n* = 8 for each group. (**D**–**F**) The MG contents (fold increase vs. that of C57 mice) of plasma, cortex, and hippocampus were measured by using the HPLC-UV method, *n* = 7–10 for each group. The data are presented as means ± SEM. # *p* < 0.05, ## *p* < 0.01 versus C57 group; * *p* < 0.05 versus KK-Ay group.

The pathogenic role of AGEs in the development and progression of cognitive deficits in diabetic subjects and animals has been widely recognized [8,9,10,18,19]. To explore the underlying mechanism of PHPB on cognitive deficits, we evaluated the levels of AGEs and MG in the plasma or brain of KK-Ay mice. The levels of AGEs and MG were markedly higher in KK-Ay mice versus C57BL/6J mice. Treatment with PHPB (150 mg/kg) could obviously reduce the generation of AGEs in plasma by 15% (*p* < 0.05) and in the hippocampus by 15% (*p* < 0.05), suppress MG accumulation in plasma by 31% (*p* < 0.05) and in the hippocampus by 20% (*p* < 0.05), respectively, in KK-Ay mice. The rosiglitazone treatment group also significantly reduced the generation of AGEs in plasma by 13% (*p* < 0.05) and in the hippocampus by 18% (*p* < 0.05), respectively, but there was no difference in MG contents relative to KK-Ay mice (Figure 5).

**Figure 6:**
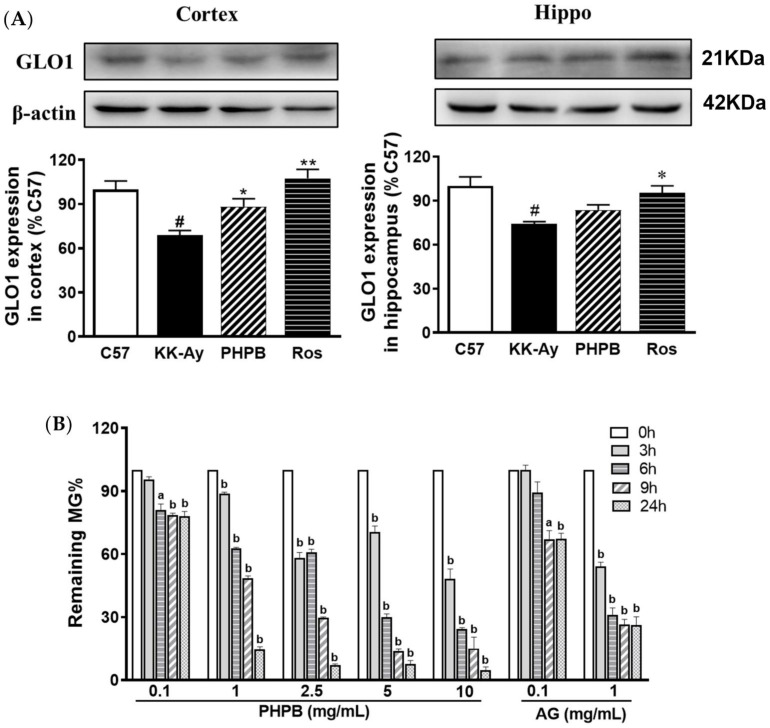
PHPB attenuated MG through up-regulating GLO1 protein and enhancing MG trapping. (**A**) Western blotting was used to detect the protein expressions of GLO1 in the cortex and hippocampus of mice treated with PHPB (150 mg/kg) or rosiglitazone (Ros, 5 mg/kg) for two months, and densitometric quantification of them, *n* = 8 for each group. (**B**) MG trapping of PHPB was detected by the HPLC method. MG (1 mmol/L) was incubated with vehicle, PHPB (0.1, 1.0, 2.5, 5.0 and 10.0 mg/mL), or aminoguanidine (AG, 0.1 mg/mL or 1.0 mg/mL) at 37 °C for 0, 3, 6, 9, and 24 h, *n* = 3 independent experiments. The data are presented as means ± SEM. # *p* < 0.05 versus C57 group; * *p* < 0.05, ** *p* < 0.01 versus KK-Ay group, ^a^ *p* < 0.05, ^b^ *p* < 0.01 versus corresponding vehicle value.

The previous studies showed that GLO1 could mediate the clearance of MG and subsequently inhibit the generation of AGEs, and protein expression of GLO1 was decreased in diabetic animals and subjects [20,21]. In the present study, the GLO1 protein was detected in the cortex and hippocampus by Western blotting. The results showed that the expression of GLO1 was obviously reduced by 31% (*p* < 0.05) and 26% (*p* < 0.05) in the cortex and hippocampus of KK-Ay mice, respectively, compared with that of C57BL/6J mice, the level of which was reversed in PHPB (increased by 28% in the cortex, *p* < 0.05) or rosiglitazone (increased by 56% in the cortex, *p* < 0.01; and 29% in the hippocampus, *p* < 0.05) treatment group (Figure 6A).

These results indicated that a possible mechanism of PHPB attenuating AGEs accumulation involved up-regulating the expression of GLO1 to enhance MG clearance.

### 2.4. Effect of PHPB on MG Clearance by MG Trapping In Vitro

In the present study, we further investigated whether PHPB could directly trap MG in vitro system. The results showed that incubation of MG with PHPB and aminoguanidine led to a decreased amount of MG in a time- and dose-dependent manner (Figure 6B). PHPB and aminoguanidine at 1 mg/mL for 24 h were found to trap MG by 93% and 48%, respectively. These values indicated that PHPB has a stronger MG trapping ability than that aminoguanidine.

### 2.5. Effects of PHPB on the SIRT1 Deacetylase Activity and Insulin Signaling Pathway in the KK-Ay Mice

**Figure 7:**
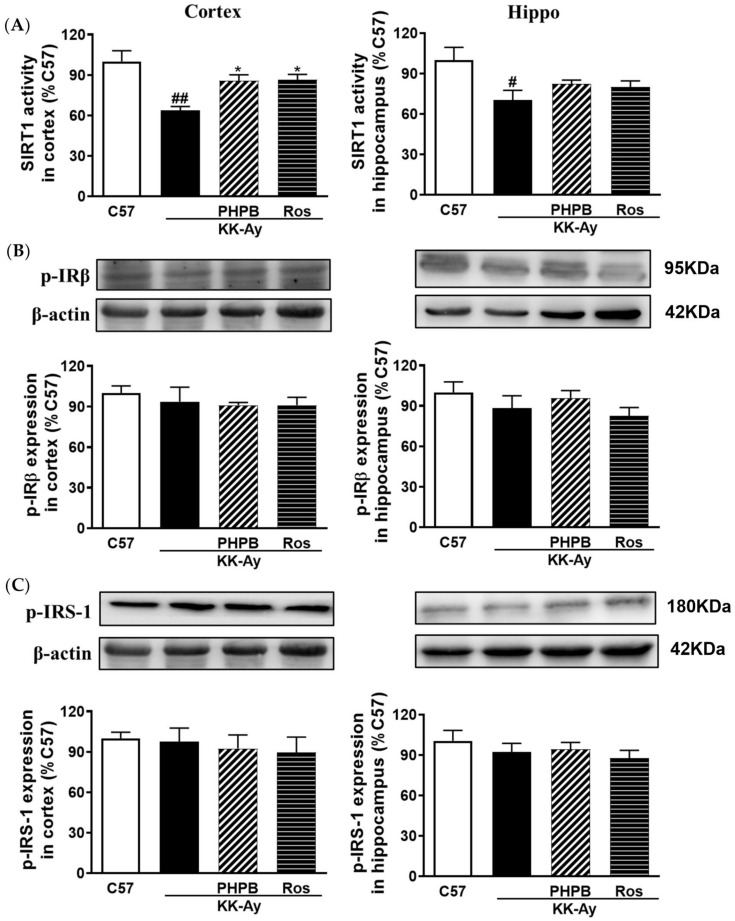

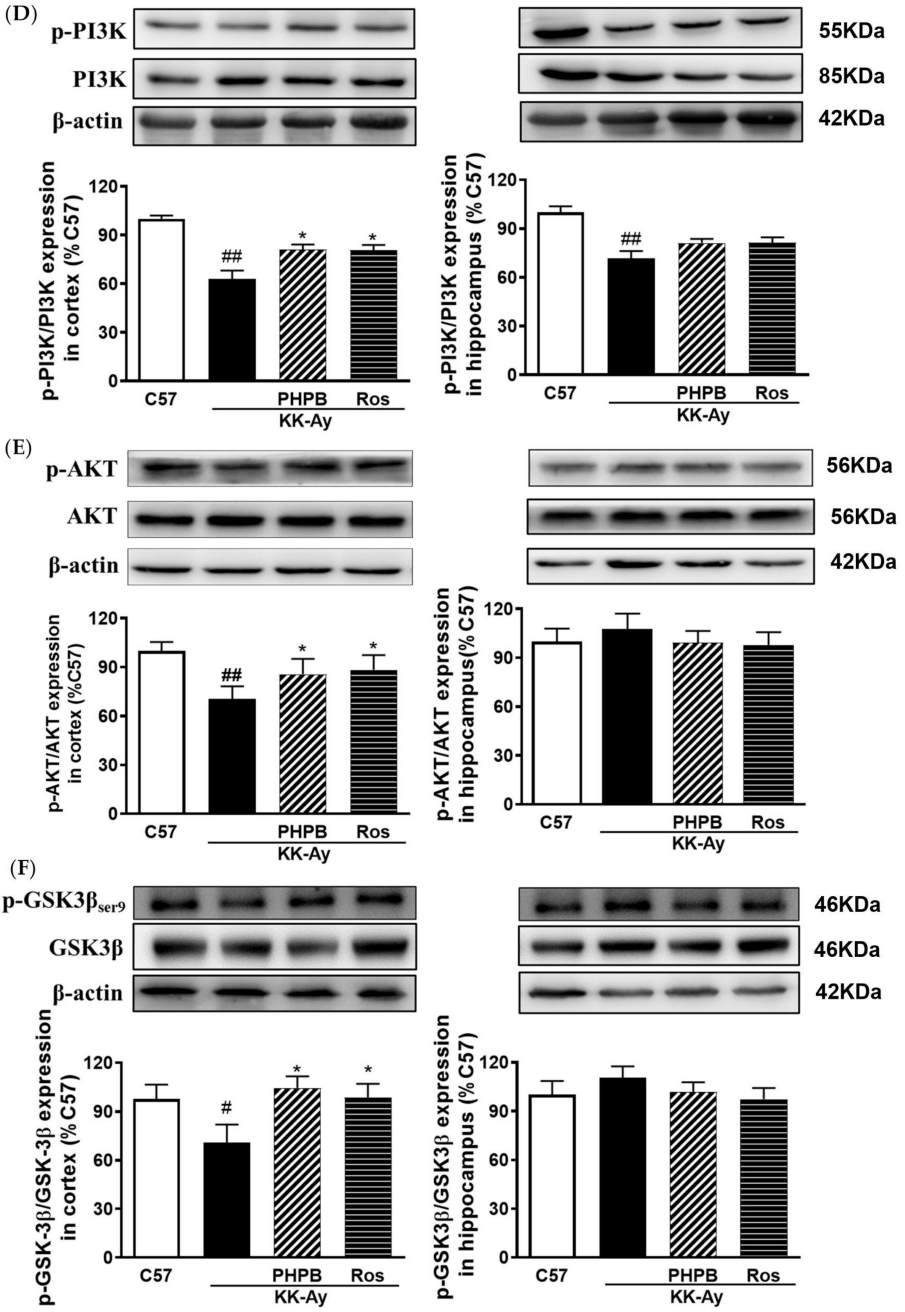
PHPB modulated SIRT1 deacetylase activity and insulin signaling pathway in cortex and hippocampus of KK-Ay mice. (**A**) The SIRT1 deacetylase activity (fold increase vs. that of C57 mice) was measured by an enzymatic fluorescence method using commercial kits. (**B**–**F**) Phosphorylation of IRβ_Tyr1150/1151_, IRS-1_Tyr859_, and PI3Kp85α or PI3K_Tyr458/p55 Tyr199_; Akt or phosphorylated Akt_Ser473_; and GSK3β or phosphorylated GSK3β_Ser9_ were determined with corresponding antibodies by western blotting in the cortex and hippocampus of mice treated by PHPB (150 mg/kg) or rosiglitazone (Ros, 5 mg/kg), and densitometric quantification of them, *n* = 5–8 for each group. The data are presented as means ± SEM. # *p* < 0.05, ## *p* < 0.01 versus C57 group; * *p* < 0.05 versus KK-Ay group.

AGEs are known to contribute to insulin resistance by down-regulation of the SIRT1 activity, an NAD+-dependent deacetylase involved in multiple physiological functions [8,9]. The SIRT1 deacetylase activity was detected in the cortex and hippocampus of KK-Ay mice. In our study, the SIRT1 deacetylase activity was significantly decreased by 36% (*p* < 0.01) and 30% (*p* < 0.05) in the cortex and hippocampus of KK-Ay mice, respectively, whereas treatment with PHPB (150 mg/kg) or rosiglitazone could significantly reverse the reduction in the cortex by 36% (*p* < 0.05) and 35% (*p* < 0.05), respectively (Figure 7A). Furthermore, we found that the expressions of p-IRβ and p-IRS were no obvious differences in the brain among all animal groups (Figure 7B,C). However, the expressions of p-PI3K, p-Akt, and p-GSK3β in the brain were down-regulated in KK-Ay mice, but PHPB (150 mg/kg) or rosiglitazone treatments can restore the above variations in the cortex (*p* < 0.05) (Figure 7D–F). These results suggested that PHPB could improve insulin signaling in the brain by increasing SIRT1 deacetylase activity, which might contribute to improving cognitive impairment.

### 2.6. Effects of PHPB on Oxidative Stress and Mitochondrial Function in KK-Ay Mice

**Figure 8:**
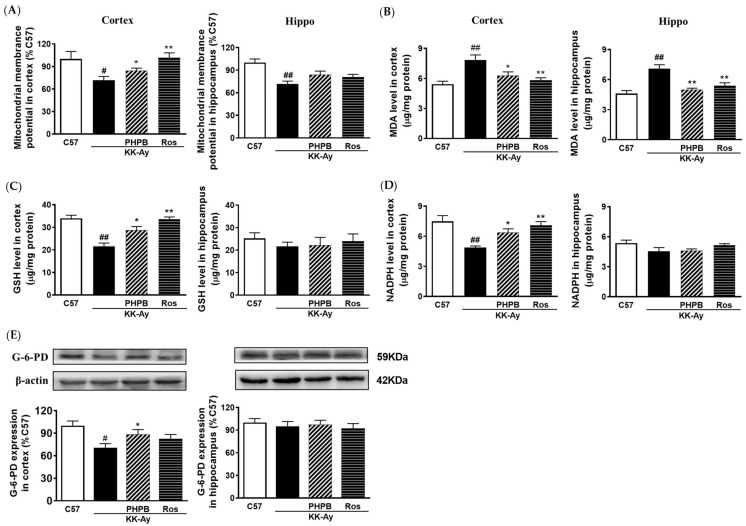
PHPB restored mitochondrial function and attenuated oxidative stress in KK-Ay mice. (**A**) Mitochondrial membrane potential (MMP, fold increase vs. that of C57 mice) was detected by JC-1 dye using commercial kits. (**B**) MDA levels and (**C,D**) the production of GSH or NADPH in the cortex or hippocampus were quantified using commercial assay kits. (**E**) Western blotting was used to detect the protein expressions of glucose-6-phosphate dehydrogenase (G-6-PD) in the cortex or hippocampus of mice treated with PHPB (150 mg/kg) or rosiglitazone (Ros, 5 mg/kg) for two months, and densitometric quantification of them. *n* = 8 for each group. The data are presented as means ± SEM. # *p* < 0.05, ## *p* < 0.01 versus C57 group; * *p* < 0.05, ** *p* < 0.01 versus KK-Ay group.

AGEs could induce oxidative stress via mitochondrial activation, thereby depleting the antioxidant defense system such as glucose-6-phosphate dehydrogenase (G-6-PD), reduced nicotinamide adenine dinucleotide phosphate (NADPH), and reduced glutathione (GSH). To better understand the effects on oxidative stress in the brain improved by PHPB inhibiting AGEs generation, we detected the protein expression of G-6-PD and the levels of malondialdehyde (MDA), NADPH, GSH, and mitochondrial membrane potential (MMP) in cortex and hippocampus. The results showed that oxidative stress was significantly increased in KK-Ay mice, evidenced by a decrease in the production of GSH (36% in cortex, *p* < 0.01) and NADPH (37% in cortex, *p* < 0.01), a drop in expression of G-6-PD (30% in cortex, *p* < 0.05), a loss of MMP (29% in the cortex, *p* < 0.05; 28% in the hippocampus, *p* < 0.01), and an elevation in MDA contents (44% in the cortex, *p* < 0.01; 54% in the hippocampus, *p* < 0.01) (Figure 8). PHPB (150 mg/kg) or rosiglitazone treatment significantly increased the production of GSH (33% for PHPB, *p* < 0.05; 56% for Ros, *p* < 0.01) and NADPH (31% for PHPB, *p* < 0.05; 46% for Ros, *p* < 0.01), reversed MMP (20% for PHPB, *p* < 0.05; 42% for Ros, *p* < 0.01), and up-regulated the protein expression of G-6-PD (25% for PHPB, *p* < 0.05) in the cortex of KK-Ay mice (Figure 8A,C–E). Meanwhile, the elevated MDA was markedly decreased in the cortex (20% for PHPB, *p* < 0.05; 36% for Ros, *p* < 0.01) and hippocampus (30% for PHPB, *p* < 0.01; 24% for Ros, *p* < 0.01) of KK-Ay mice treated by PHPB (150 mg/kg) or rosiglitazone (Figure 8B). These results indicated that PHPB could improve mitochondrial function and reduce oxidative stress in the brain, possibly by inhibiting the generation of AGEs.

## 3. Discussion

T2DM and AD are both age-related diseases that affect millions of people worldwide. Broadly supported by epidemiological data, the higher incidence of AD among type 2 diabetic patients led to T2DM being considered a tangible risk factor for AD onset, known as DE, compared to the normal subjects [2,3,4,22,23]. The onset and progression of DE are complex and involve many factors; AGEs may be an important mechanism of DE [5,7,8,9,10]. The accumulation of protein glycation was also detected in the cerebrospinal fluid of individuals with AD as compared with control subjects [24,25]. Dysfunction of brain glucoregulation and the increase of cerebral-glucose influx might be key reasons for AGEs accumulation [26]. In recent years, a few studies showed that cerebral-glucose levels were significantly elevated in diabetic animals, such as STZ-induced diabetes rat model and obsess monogenic rodent models of spontaneous T2DM such as Zucker rats and BB/Wor diabetic Sprague-Dawley rats [27]. The elevation of cerebral-glucose in the central nervous system (CNS) results in neurotoxicity that can lead to an increase in the incidence of AD [27,28,29]. A previous study showed that spontaneously obese KK-Ay mice exhibited typical characteristics of T2DM with obvious cognitive impairment and were considered a reliable animal model for studying DE [30]. In the current study, our data suggested, for the first time, PHPB cloud improves diabetes-induced cognitive impairment in type 2 diabetic KK-Ay mice probably via inhibiting the generation of the AGEs and subsequently modulating SIRT1/insulin signaling pathway and reducing oxidative stress.

In the present study, KK-Ay mice displayed cognitive impairment, with glucose elevation, AGEs accumulation, oxidative stress, insulin resistance, reducing the contents of GLO1 protein, NADPH, or GSH, and MG (a major precursor of AGEs) hyperactivity in the brain or plasma. It is similar to the reports in AD patients and animal models [25,31]. PHPB intervention significantly improved diabetes-induced cognitive impairment without obvious differences in body weight gain and levels of plasma-glucose, cerebral-glucose and plasma-insulin. Rosiglitazone also exhibited obvious improvement in cognitive impairment, with an increase in body weight, enhancement of insulin sensitivity, and reduction of glucose levels in the brain or plasma. These results indicated that the underlying mechanism of PHPB in preventing cognitive impairment might be distinct from those of rosiglitazone without relevance to cerebral-glucose reduction.

AGEs might play a more important role than hyperglycemia because the inhibition of glycoxidation products could prohibit the onset and progression of diabetic complications without alteration in glycemia [32]. Accumulated studies proved that the AGEs were involved in the onset and progression of chronic diseases, such as diabetes, cerebrovascular and cardiovascular diseases, neurological disorders, and so on, as well as the senescence processes [8,9,10]. The excessive accumulation of AGEs downregulates PI3K/Akt/GSK-3β signaling pathway by inhibiting SIRT1 expression, promotes the astrocytic differentiation of cultured neurospheres by inhibiting neurogenesis through the Notch-Hes1 pathway, increases expression of inflammatory genes, and decreases learning and memory function [33,34]. Our previous research also proved that PHPB could significantly improve cognitive function in type 2 diabetic KK-Ay mice via elevation of neurotrophic factor and vesicular glutamate transporter 1 (vGLUT1), inhibition of neuroinflammatory, and modulation of PI3K/Akt/GSK-3β signaling pathway [14]. These results suggest that PHPB may be associated with the reduction of AGEs production in the treatment of DE. In the present study, we further detected AGEs generation, SIRT1/insulin signaling pathway and oxidative stress in the plasma or brain of type 2 diabetic KK-Ay mice.

Our results showed that KK-Ay mice treated with PHPB decreased the generation of AGEs or MG. Rosiglitazone as a positive control also showed similar effects, consistent with existing reports that rosiglitazone inhibits the formation of AGEs in the hippocampus and cortex of db/db mice [15]. Thus, the inhibition of AGEs generation by PHPB may largely contribute to the amelioration of DE. Although the effects of PHPB in AGEs generation were confirmed, the precious molecular mechanism remains to be further elucidated. Glucose flux leads to the formation of the highly reactive dicarbonyl metabolite, such as MG [35]. MG is known as the main precursor of AGEs, which is mainly formed by the degradation of triose phosphate produced during glycolysis [35]. Therefore, therapeutic strategies that seek to reduce MG or enhance its clearance and subsequently inhibit AGEs generation may effectively prevent the progression of DE. With GSH as a cofactor, GLO-1 plays a key role in the detoxification of MG in the human body, promotes the clearance of MG, and directly inhibits the generation of AGEs [10]. GLO-1 deficiency is associated with abnormally elevated serum AGEs levels in a hemodialysis patient [36]. GLO-1 overexpression in rats with streptozotocin (STZ)-induced diabetes reduces hyperglycemia-induced levels of carbonyl stress, AGEs, and oxidative stress [37]. In addition, the activity and protein expression of GLO-1 have marked down-regulation in the cortex or hippocampus in STZ-induced diabetic rats, coupled with GSH reduction and AGEs accumulation [21]. In the present study, PHPB or rosiglitazone reversed the reduction of GLO-1 protein and GSH contents in the brain of KK-Ay mice. In vitro, the MG trapping assay also demonstrated PHPB could clear MG from plasma or the brain in a dose-dependent manner. However, our previous results showed that PHPB was rapidly (within 20 min) and completely converted into its active form (3-n-butylphthalide) after treatment in rats and dogs [11], implying that directly trapping MG was not a main way for PHPB to inhibit the generation of AGEs. Overall, these demonstrated that PHPB mediated-suppression of AGEs accumulation might be through up-regulation of GLO1 protein and partly enhancement of MG trapping.

Oxidative stress and insulin resistance are coexisting characteristics of diabetes, and both are considered key prompting factors of DE [3,37,38]. In T2DM, insulin resistance leads to increase glucose levels, which causes oxidative stress. Subsequently, elevated glucose promotes the production of AGEs, which further triggers insulin resistance and the generation of reactive oxygen species (ROS) [3,22]. ROS generated by AGEs via mitochondrial activation induces oxidative stress, thereby depleting NADPH, which is a major cofactor of antioxidant enzyme systems [39]. On the other hand, AGEs bind to the receptor for AGE (RAGE), thereby activating various signaling cascades including inflammatory response nuclear transcription factor (NF)-κB and downstream insulin factors PI3K/Akt [3,26]. Insulin signaling is known to be disrupted by AGEs via down-regulation of the SIRT1 protein [26]. Our previous results showed that PHPB could inhibit oxidative stress or inflammatory response and modulate PI3K/Akt signaling pathways [13,14]. Some studies have shown that increased activity of GSK-3β directly leads to hyperphosphorylation of tau, which contributes to the formation of NFTs that destabilize microtubule integrity [40]. In agreement with these results, here, PHPB exhibits antioxidation activity evidenced by elevating G-6-PD protein expressions, promoting the production of GSH and NADPH, restoring mitochondrial function, increasing ATP contents, and reducing MDA levels in the brain of KK-Ay mice. Also, PHPB treatment also improved the insulin signaling pathway by increasing SIRT1 deacetylase activity and activating the PI3K/Akt/GSK3β signaling pathway.

Although rosiglitazone has also shown similar or stronger beneficial effects for DE, its use has been limited because of concerns about body weight gain and cardiovascular toxicity [41]. PHPB was considered to be a good tolerance and safe agent [11], which might be developed into a potential therapeutic agent for DE.

The limitations of this study consist of the sole animal model, dietary differences (the high-fat diet in KK-Ay Mice and the standard diet in C57 mice), and the single behavioral assessment in learning and memory. However, PHPB has been proven the efficacy in improving diabetes-induced cognitive impairment in KK-Ay mice [13,14]. In the present study, PHPB significantly shortened escape latency during the 6-day acquisition training in KK-Ay mice, whereas it was no significant difference in the probe trial test of the Morris water maze. Thus, further investigations are still needed to validate the neuroprotective effects of PHPB against other animal models of diabetes (e.g., db/db mice, streptozotocin-induced diabetic rats, etc.) through more behavioral tests (for example, Y Maze, etc.), and characterize potential molecular mechanisms.

## 4. Materials and Methods

### 4.1. Animals and Drug Administration

Male KK-Ay mice (*n* = 69, 3-month-old) and age-matched male C57BL/6J mice (*n* = 10) were purchased from HFK Bioscience Co. Ltd. (Beijing, China). KK-Ay mice were fed a high-fat diet. Age-matched male C57BL/6J mice fed with the standard chow diet were employed as non-diabetic control by oral gavage distilled water. Male KK-Ay mice were randomly divided into four groups: untreated group (*n* = 19) and treated groups. Treated groups received PHPB (50 mg/kg/day, *n* = 17 and 150 mg/kg/day, *n* = 17, Yunnan Haobang Pharmaceutical Co. Ltd., Yunnan, China) [13] or Rosiglitazone (5 mg/kg/day, *n* = 16, Chongqing Taiji Pharmaceutical Co., Ltd., Chongqing, China) [15,42] by oral gavage. All treatment continued for two months, beginning at 3-month-old, and body weight was monitored throughout the experiment.

During the experimental period, animals were allowed free access to food and water ad libitum in a temperature-controlled environment of 23 ± 2 °C and maintained at a 12-h light/dark cycle per day. All experiments were approved and performed in accordance with the guidelines for the care and use of laboratory animals and were approved by the Animal Care Committee of the Peking Union Medical College and the Chinese Academy of Medical Sciences (Beijing, China) (Approval number 00000634).

### 4.2. Morris Water Maze Test

The Morris water maze (MWM) task was used to detect the spatial learning and memory ability of mice. The water maze was situated in a quiet room and composed of a circular white pool (120 cm in diameter and 50 cm in height), filled with water made opaque by the addition of milk powder. As described in reference [30], the depth of water was 30 cm, and the temperature of the water was 23 ± 1 °C. The pool was divided into four quadrants with a transparent acrylic platform (10 cm in diameter, hidden below the water surface 1.0 cm) placed at the center of the southwest quadrant. During the water maze, the orientation navigation test was performed blindly for six consecutive days with the location of the platform fixed, but the starting positions were changed every day. The mice were trained twice a day, from 8:00 to 12:00 a.m. and from 13:00 to 17:00 p.m. Each mouse was placed in the pool and allowed 120 s to find the hidden platform and needed to remain there for 30 s; the escape latency was recorded. If the mouse was not able to reach the platform, the experimenter helped the mouse stay on it for 30 s. As for the failed trainer, the escape latency was considered 120 s. On the seventh day, the platform was removed, and the first latency time and times of crossings over the original platform location and swim speed were recorded to assess the spatial memory maintenance/motor ability without the influence of chance encounters with the platform. The mice with blindness were excluded via the visible platform test.

### 4.3. Metabolic Determination

After 2 months of treatment, the levels of fasting blood glucose (FBG), lipid, and insulin were determined in KK-Ay mice and C57BL/6J mice, as previously described [13,14,30]. Briefly, blood was collected from the tail vein after fasting for 2 h in mice. FBG levels were measured by the glucose oxidase method, and contents of plasma triglycerides (TG, Lot: 218081) or total cholesterol (TC, Lot: 212072) were determined by enzymatic colorimetric methods using commercial kits (BioSino Inc., Beijing, China). The concentration of plasma insulin was detected by ELISA assay kit (80-INSMS-E01, E10, Alpco. Inc., Salem, NH, USA).

### 4.4. Microdialysis

#### 4.4.1. Surgery

Mice were anaesthetized using isoflurane (95% in O_2_ for induction, 1.5% for maintenance; IsoFlo^®^, Abbott Park, IL, USA.) and placed in a stereotaxicframe (RWD Life Science Co., Ltd., Shenzhen, China). Then an intracranial guide (CMA/Microdialysis, Stockholm, Sweden) was implanted into the hippocampus (−2.18 mm from Bregma, −2.85 mm from midline, 2.35 mm from dorsal surface of brain), as described previously [43,44]. After surgery, guide cannula obturators were replaced by microdialysis probes (CMA7; membrane length: 1 mm theoretical cutoff: 6000 Da; CMA/Microdialysis, Solna, Sweden), and mice were recovered for 24 h in their home cages with access ad libitum to food and water.

#### 4.4.2. Tissue Microdialysis

24 h following recovery, mice were anaesthetized using isoflurane, and another microdialysis probes were implanted into the femoral artery. Then microdialysis probes were continuously perfused with artificial cerebrospinal fluid (aCSF, 147 mM NaCl, 3 mM KCl, 1 mM MgCl_2_·6H_2_O, 0.787 mM CaCl_2_·6H_2_O, 186 μM Ascorbate, 3.52 mM NaH_2_PO_4_·H_2_O, pH 7.4) at the work flow-rate of 1 μL/min (CMA/400 microdialysis pump, Solna, Sweden) [43]. The microdialysis aliquots were collected during the final 60 min of the 2-h study period when the experiment had reached steady-state concentrations of the substrates. Dialysate glucose was measured by the glucose oxidase method.

### 4.5. Tissue Processing

After behavioral or microdialysis testing, animals were anaesthetized using isoflurane. The arterial blood of the mice was collected in a tube coated with EDTA, and plasma was separated by centrifugation at 1000× *g* for 10 min at 4 °C. The brains of the mice were harvested and immediately weighed. The cortex and hippocampus were rapidly dissected via surgery on ice.

### 4.6. Biochemical Analysis

Tissue treatment was performed according to the manufacturer’s instructions. The levels of adenosine triphosphate (A095-1-1, ATP), malondialdehyde (A003-1-2, MDA), and reduced glutathione (A006-2-1, GSH) in the cortical and hippocampal homogenate of the animals were determined according to the manufacturers’ instructions (Nanjing Jiancheng Bioengineering Research Institute, Nanjing, China). The content of reduced nicotinamide adenine dinucleotide phosphate (NADPH) was quantified using a NADP^+^/NADPH assay kit (ab65349, Abcam, Cambridge, UK). The concentrations of AGEs in plasma and tissue were measured using an AGEs ELISA kit (STA-317, Cell Biolabs Inc., San Diego, CA, USA) following the manufacturer’s instructions. SIRT1 activity was detected by the SIRT1 assay kit (CS1040, Sigma, St Louis, MO, USA). The homogenate protein levels were quantified using a BCA protein assay kit (P1511, Applygen Technologies Inc., Beijing, China), as outlined previously [43].

### 4.7. Methylglyoxal Measurement

The amount of MG was measured by a specific and sensitive HPLC-UV method, as described before, with some modifications [45,46]. Briefly, the supernatant of brain homogenate or plasma was incubated in the dark for 24 h with 0.5 N perchloric acid (30755, PCA, Sigma, St Louis, MO, USA), 1 mM o-phenylenediamine (P23938, O-PD, derivatizing agent, Sigma, St Louis, MO, USA), and 25 μM 5-methylquinoxaline (W320307, 5-MQ, internal standard, Sigma, St Louis, MO, USA) at room temperature. The samples were further centrifuged at 13,000× *g* for 10 min, and the supernatants were filtered with a 0.22 μm membrane before addition to the HPLC sample vials. MG was quantified using an Agilent 1100 high-performance liquid chromatography (HPLC) system (Palo Alto, CA, USA) via a Dikma Diamonsil C18 column (4.6 × 250 mm, 5 μm). Acetonitrile (34851, 40%, Sigma, St Louis, MO, USA) was kept for running the samples. Each sample was run for 15 min with a flow rate of 1.0 mL/min. Other analysis conditions were as follows: detector wavelength, 315 nm; sample injection volume, 20 μL.

### 4.8. Determination of Methylglyoxal (MG) Trapping of PHPB In Vitro

MG (M0252, 1.0 mmol/L; Sigma, St Louis, MO, USA) was incubated with vehicle (PBS) or PHPB (0.1, 1.0, 2.5, 5.0, and 10.0 mg/mL) at 37 °C for 0, 3, 6, 9, and 24 h. Aminoguanidine (396494, AG, 0.1 mg/mL or 1.0 mg/mL; Sigma, St Louis, MO, USA) was used as positive controls. The amounts of MG in samples with different treatments were measured by the HPLC analysis described above. The MG trapping ratio was calculated as the percentage of remaining MG.

### 4.9. Western Blotting

Tissue lysates were prepared as described earlier [17,18] and the protein concentration in the supernatant was determined by the BCA protein assay. Aliquots of tissue lysates (40 μg of protein each) were separated on 7.5–10% SDS-PAGE, electrotransferred to a polyvinylidene difluoride (PVDF) membrane (Bio-Rad Laboratories Co., Ltd., Hercules, CA, USA), blocked with 5% nonfat milk in TBS-Tween buffer for 1.5 h at room temperature, and incubated overnight at 4 °C with the primary antibody, G-6-PD (ab933, Abcam, Cambridge, UK), phosphorylated Tau_Ser404_ (#20194), phosphorylated GSK3β_Ser9_ (#5558), GSK3β (#12456), phosphorylated IRS-1_Tyr859_ (#3070), Akt (#4685), phosphorylated Akt_Ser473_ (#4060), PI3Kp85α (#13666), and phosphorylated PI3K_Tyr458/p55 Tyr199_ (#17366) (Cell Signaling Technology, Inc., Danvers, MA, USA), GLO1 (SC-133144) and phosphorylated-insulin Rβ_Tyr 1150/1151_ (SC-81500) (Santa Cruz Biotechnology, Inc., CA, USA), β-actin (ab8224, Abcam, Cambridge, UK), and then with horseradish peroxidase-conjugated secondary antibody (Santa Cruz Biotechnology, Inc., CA, USA) for 1 h at room temperature. After extensive washing, the immunoreactive proteins were detected with an Enhanced Chemiluminescence Detection System (ECL; Fujifilm, Tokyo, Japan) and analyzed by densitometric evaluation using Image J software (V1.8.0.112). The values were normalized to β-actin intensity levels. Alterations in cortical and hippocampal protein expression were presented as fold-changes relative to age-matched C57BL/6J mice.

### 4.10. Measurement of Mitochondrial Membrane Potential (MMP)

Mitochondria were isolated by mitochondrial isolation assay kit (G006-1-1), and MMP was detected using an MMP assay kit with JC-1 dye (G0069-1-3) according to the manufacturer’s protocol (Nanjing Jiancheng Bioengineering Research Institute; Nanjing, China). The mitochondrial protein levels were quantified using a BCA protein assay kit (P1511, Applygen Technologies Inc., Beijing, China).

### 4.11. Statistical Analysis

Statistical tests were performed by SPSS version 16.0 software. All the data are expressed as mean ± SEM. Data normality was verified by Shapiro–Wilk test. The intergroup variations on the indexes of the water maze positioning navigation trial were analyzed by two-way analysis of variance (ANOVA) with repeated determinations. The intergroup variations of the water maze probe trial, biochemistry, and semi-quantitative analysis of western blotting were analyzed by one-way ANOVA, followed by post hoc LSD or Dunnett’s test. *A p*-value of less than 0.05 was statistically significant.

## 5. Conclusions

Our results indicate that PHPB is effective in modulating SIRT1/insulin signaling pathway and reducing oxidative stress via inhibiting the generation of the AGEs, which supports its application as a promising therapeutic for the prevention and treatment of DE in the future.

## Data Availability

Data are contained within the article.
